# Supplementary material for: Kinematic alteration in three-dimensional reaching movement in C3-4 level cervical myelopathy
Source: PLoS One. 2023 Nov 30;18(11):e0295156. doi: 10.1371/journal.pone.0295156 (PMC10688652; doi:10.1371/journal.pone.0295156)
Supplement: Attachment — Submitted filename: Response to Reviewers.docx [file pone.0295156.s002.docx]

Reply to the editors’ suggestions and comments.

Thank you very much for your comments and suggestions.

Changes of highlight with yellow color in the marked-up manuscript.

Journal Editor

Journal requirements:

When submitting your revision, we need you to address these additional requirements.

Thank you for your comments and suggestions. Your comments and suggestions encouraged us to improve our manuscript.

1. Please ensure that your manuscript meets PLOS ONE's style requirements, including those for file naming. The PLOS ONE style templates can be found at

https://journals.plos.org/plosone/s/file?id=wjVg/PLOSOne_formatting_sample_main_body.pdf and

https://journals.plos.org/plosone/s/file?id=ba62/PLOSOne_formatting_sample_title_authors_affiliations.pdf

Thank you for your suggestions. According to the PLOS ONE style templates, we changed the figure file formats and position of page numbers, deleted empty rows in tables, and revised references.

2. Note from Emily Chenette, Editor in Chief of PLOS ONE, and Iain Hrynaszkiewicz, Director of Open Research Solutions at PLOS: Did you know that depositing data in a repository is associated with up to a 25% citation advantage (https://doi.org/10.1371/journal.pone.0230416)? If you’ve not already done so, consider depositing your raw data in a repository to ensure your work is read, appreciated and cited by the largest possible audience. You’ll also earn an Accessible Data icon on your published paper if you deposit your data in any participating repository (https://plos.org/open-science/open-data/#accessible-data).

Thank you for your suggestion. After careful consideration, we have determined to submit a data set as a supporting information file.

3. Thank you for stating the following financial disclosure:

“The funders had no role in study design, data collection and analysis, decision to publish, or preparation of the manuscript.”

At this time, please address the following queries:

a) Please clarify the sources of funding (financial or material support) for your study. List the grants or organizations that supported your study, including funding received from your institution.

b) State what role the funders took in the study. If the funders had no role in your study, please state: “The funders had no role in study design, data collection and analysis, decision to publish, or preparation of the manuscript.”

c) If any authors received a salary from any of your funders, please state which authors and which funders.

d) If you did not receive any funding for this study, please state: “The authors received no specific funding for this work.”

Please include your amended statements within your cover letter; we will change the online submission form on your behalf.

Thank you for your comments. Our study was supported by JSPS KAKENHI under Grant number 21K17507. Moreover, the funders had no role in our study design, data collection and analysis, decision to publish, or preparation of the manuscript. In order to clarify this, we added the funding information in the cover letter.

4. We note that you have indicated that data from this study are available upon request. PLOS only allows data to be available upon request if there are legal or ethical restrictions on sharing data publicly. For more information on unacceptable data access restrictions, please see http://journals.plos.org/plosone/s/data-availability#loc-unacceptable-data-access-restrictions.

In your revised cover letter, please address the following prompts:

a) If there are ethical or legal restrictions on sharing a de-identified data set, please explain them in detail (e.g., data contain potentially sensitive information, data are owned by a third-party organization, etc.) and who has imposed them (e.g., an ethics committee). Please also provide contact information for a data access committee, ethics committee, or other institutional body to which data requests may be sent.

b) If there are no restrictions, please upload the minimal anonymized data set necessary to replicate your study findings as either Supporting Information files or to a stable, public repository and provide us with the relevant URLs, DOIs, or accession numbers. For a list of acceptable repositories, please see http://journals.plos.org/plosone/s/data-availability#loc-recommended-repositories.

We will update your Data Availability statement on your behalf to reflect the information you provide.

Thank you for your suggestion. According to your instructions, we would like to have prepared a data set as a supporting information file and submitted it.

5. PLOS requires an ORCID iD for the corresponding author in Editorial Manager on papers submitted after December 6th, 2016. Please ensure that you have an ORCID iD and that it is validated in Editorial Manager. To do this, go to ‘Update my Information’ (in the upper left-hand corner of the main menu), and click on the Fetch/Validate link next to the ORCID field. This will take you to the ORCID site and allow you to create a new iD or authenticate a pre-existing iD in Editorial Manager. Please see the following video for instructions on linking an ORCID iD to your Editorial Manager account: https://www.youtube.com/watch?v=_xcclfuvtxQ

Thank you for your suggestion. According to your instruction, we ensure the ORCID information of the corresponding author.

6. We note that Figure 1 in your submission contain copyrighted images. All PLOS content is published under the Creative Commons Attribution License (CC BY 4.0), which means that the manuscript, images, and Supporting Information files will be freely available online, and any third party is permitted to access, download, copy, distribute, and use these materials in any way, even commercially, with proper attribution. For more information, see our copyright guidelines: http://journals.plos.org/plosone/s/licenses-and-copyright.

We require you to either (1) present written permission from the copyright holder to publish these figures specifically under the CC BY 4.0 license, or (2) remove the figures from your submission:

a. You may seek permission from the original copyright holder of Figure 1 to publish the content specifically under the CC BY 4.0 license.

We recommend that you contact the original copyright holder with the Content Permission Form (http://journals.plos.org/plosone/s/file?id=7c09/content-permission-form.pdf) and the following text:

“I request permission for the open-access journal PLOS ONE to publish XXX under the Creative Commons Attribution License (CCAL) CC BY 4.0 (http://creativecommons.org/licenses/by/4.0/). Please be aware that this license allows unrestricted use and distribution, even commercially, by third parties. Please reply and provide explicit written permission to publish XXX under a CC BY license and complete the attached form.”

Please upload the completed Content Permission Form or other proof of granted permissions as an "Other" file with your submission.

In the figure caption of the copyrighted figure, please include the following text: “Reprinted from [ref] under a CC BY license, with permission from [name of publisher], original copyright [original copyright year].”

b. If you are unable to obtain permission from the original copyright holder to publish these figures under the CC BY 4.0 license or if the copyright holder’s requirements are incompatible with the CC BY 4.0 license, please either i) remove the figure or ii) supply a replacement figure that complies with the CC BY 4.0 license. Please check copyright information on all replacement figures and update the figure caption with source information. If applicable, please specify in the figure caption text when a figure is similar but not identical to the original image and is therefore for illustrative purposes only.

Thank you for your comments. Indeed, we own the electronic-mechanical whack-a-mole-type task shown in Figure 1 and photographed it and created the Figure ourselves. Therefore, Figure 1 does not contain any copyright, and there is no problem if it is used by third parties.

7. Please review your reference list to ensure that it is complete and correct. If you have cited papers that have been retracted, please include the rationale for doing so in the manuscript text, or remove these references and replace them with relevant current references. Any changes to the reference list should be mentioned in the rebuttal letter that accompanies your revised manuscript. If you need to cite a retracted article, indicate the article’s retracted status in the References list and also include a citation and full reference for the retraction notice.

Thank you for your suggestions. According to the PLOS ONE style templates, we checked and changed reference information.

Reply to the reviewer’s suggestions and comments.

Reviewers' comments:

Reviewer's Responses to Questions

Comments to the Author

5. Review Comments to the Author

Please use the space provided to explain your answers to the questions above. You may also include additional comments for the author, including concerns about dual publication, research ethics, or publication ethics. (Please upload your review as an attachment if it exceeds 20,000 characters)

Reviewer #1: The authors performed a study addressing “Kinematic alteration in three-dimensional reaching movement in C3-4 level cervical myelopathy”. However, there is one unclear area. Why is it appropriate to monitor 3D reaching movement and measure movement time (MT) and 3D movement distance (MD) during the task to have the objectives of this study achieved? Is it difficult to do so by other methods? Please describe your reasons in more detail.

Thank you very much for your comment. We found your comment was very helpful in improving our manuscript. We revised the sentence expressing the usefulness of measuring 3D reaching movement time and distance and added the reference related as follows.

Line 69-83

In order to solve this problem, several studies used an accelerometer to independently measure reaching function [13, 14]. Although the sensor makes it possible to easily measure kinematic changes during reaching movement, data obtained from only one-axis acceleration (e.g., forward-backward direction) is still not enough to understand upper limb movement in three-dimensional (3D) space [13]. Recently, several techniques have been developed for synchronizing multiple accelerometers or combining three-axis acceleration data [14], but the necessity of a special calculation program and the time cost of data analysis limit its practical use in clinical environments. Therefore, the evaluation not depending on grasping or manipulation and detecting the changes in 3D reaching movements is necessary to accurately measure proximal arm function in CM.

The purpose of the study was to examine the sole reaching function in 3D space without being affected by distal function. In order to separate proximal from distal function, we used an electronic-whack-a-mole-type task which only requires shoulder and elbow joint movements, not accompanied by forearm or finger movements. We monitored the reaching movement time and distance during the task, suggesting that these parameters can detect the changes in any direction of reaching movement. We hypothesized that patients with C3-4 compression may have decreased reaching capability and the kinematic alteration has a negative influence on the upper extremity performance in CM patients.

Line 313-318

13.	Noguchi N, Lee B, Kondo K, Ino M, Kamiya S, Yamazaki T. Changes in reaching skill in patients with cervical spondylosis after cervical decompression surgery. J Phys Ther Sci. 2019; 31(10): 760-764. doi: 10.1589/jpts.31.760.

14.	Amici C, Ragni F, Ghidoni M, Fausti D, Bissolotti L, Tiboni M. Multi-Sensor Validation Approach of an End-Effector-Based Robot for the Rehabilitation of the Upper and Lower Limb. Electronics. 2020; 9: 1751. doi.org/10.3390/electronics9111751.

Reviewer #2: The article title was so intresting to me , that made me to read all the article ,

You can move foreward and , do some future researches after this study to conplete your study on whole spine ,

Thanks

Thank you very much for your interest in our study. Your comments encouraged us to improve our manuscript and we appreciate your kind consideration.

During the process of revision, we noticed that some values in Table 2 were not located properly. We are sorry for the careless mistakes.
